# Determination of Carbohydrate Composition in Mealworm (*Tenebrio molitor* L.) Larvae and Characterization of Mealworm Chitin and Chitosan

**DOI:** 10.3390/foods10030640

**Published:** 2021-03-18

**Authors:** Yang-Ju Son, In-Kyeong Hwang, Chu Won Nho, Sang Min Kim, Soo Hee Kim

**Affiliations:** 1 Smart Farm Research Center, Korea Institute of Science and Technology, Gangneung Institute of Natural Products, Gangneung 25451, Korea; yangjuson@kist.re.kr (Y.-J.S.); cwnho@kist.re.kr (C.W.N.); kimsm@kist.re.kr (S.M.K.); 2 Department of Food and Nutrition and Research Institute of Human Ecology, Seoul National University, Seoul 08826, Korea; ikhwang@snu.ac.kr; 3 Department of Culinary Arts, Kyungmin University, Uijeongbu 11618, Korea

**Keywords:** edible insect, mealworm, sugar content, chitin, microscopy, degree of acetylation

## Abstract

Mealworm (*Tenebrio molitor* L.) is a classic edible insect with high nutritional value for substituting meats from vertebrates. While interest in mealworms has increased, the determination of carbohydrate constituents of mealworms has been overlooked. Thus, the aim of the present study was to investigate the carbohydrate content and composition of mealworms. In addition, the characteristics of mealworm chitin were determined as these were the major components of mealworm carbohydrate. The crude carbohydrate content of mealworms was 11.5%, but the total soluble sugar content was only 30% of the total carbohydrate content, and fructose was identified as the most abundant free sugar in mealworms. Chitin derivatives were the key components of mealworm carbohydrate with a yield of 4.7%. In the scanning electron microscopy images, a lamellar structure with α-chitin configuration was observed, and mealworm chitosan showed multiple pores on its surface. The overall physical characteristics of mealworm chitin and chitosan were similar to those of the commercial products derived from crustaceans. However, mealworm chitin showed a significantly softer texture than crustacean chitin with superior anti-inflammatory effects. Hence, mealworm chitin and chitosan could be employed as novel resources with unique advantages in industries.

## 1. Introduction

The global population is still increasing and, consequently, likely food shortcomings need to be addressed. Furthermore, the increasing consumption of meat in developing countries affects the sustainability issue because livestock-rearing requires excessive energy inputs [1]. Meat products are excellent protein sources for humans, and therefore, the search for substitutes has reached an impasse. Edible insects are attractive alternatives because of their high protein contents, and these show superior conversion efficiencies that are almost ten times higher than those of the ruminants [2]. Moreover, insects require less feedstuff and space, and omit fewer greenhouse gases than livestock [3]. Accordingly, the Food and Agriculture Organization (FAO), following a suggestion already made in 1975 by Meyer-Rochow [4], also identified edible insects as appropriate alternative protein sources to meats from vertebrates [5].

Among various types of edible insects, the mealworm (*Tenebrio molitor* L.) is one of the most popular species as a food and feed resource, and its extensive utilization has resulted in the advancement and establishment of commercial large-scale rearing systems [6]. Significant interest in mealworms has led to numerous studies on their growth and development [7], mass production systems [8], and nutrition value for use as feed [9,10]. In addition, the composition and nutritional value of mealworms have been verified for use in food products [11,12], and consumer perception [13] as well as industrial processing technologies have also been investigated [14,15]. However, most studies have focused on the composition of proteins or fats in mealworms, but the carbohydrate content has not been extensively studied. Although it is well known that mealworms have low carbohydrate contents, the constituents and properties of carbohydrates should be verified to further understand their characteristics. Moreover, chitin derivatives, the predicted major carbohydrates in mealworms, are important chemical compounds for functional nutrients or industrial materials.

The aim of the present study is to investigate the characteristics and composition of carbohydrate content in mealworms as it has not been extensively investigated in previous studies. To determine the carbohydrate content and its constituents, the contents of crude carbohydrate and total soluble sugar were examined, and the free sugar composition was identified. As chitin and its derivatives are the major constituents of the mealworm carbohydrate content, mealworm chitin and chitosan were extracted and prepared. Accordingly, their physical and structural properties were determined using diverse analytical methods. Thus, the analysis of carbohydrate compounds in mealworms was conducted, and the characteristics of chitin derivatives were determined to identify the possible applications of mealworm chitin.

## 2. Materials and Methods

All chemical compounds and solvents without provider information in the present study were purchased from Sigma-Aldrich Corporation (St. Louis, MO, USA).

### 2.1. Preparation of Mealworm Powder

Mealworm larvae were reared on a farm located at Gyeonggi-do, Republic of Korea, and mealworm powder was prepared according to the procedure described by Son et al. [16]. Briefly, mealworms were blanched using boiling water for 3 min (1:5, *w*/*w*), and the outer water was removed. The mealworms were dried in a hot-air dryer (LH.FC-PO-150; Lab house, Pocheon, Korea) at 60 °C for 12 h. The dried mealworms were milled using a HR-2860 blender (Philips, Amsterdam, The Netherlands). The pulverization process was repeated until all powders passed through a 535-μm diameter sieve.

### 2.2. Total Soluble Sugar and Reducing Sugar Contents

Mealworm powder (10 g) was mixed with distilled water (1:10, *w*/*v*) and extracted at 70 °C with shaking at 150 rpm for 2 h (BS-21; Jeiotech, Daejeon, Korea). The solution was centrifuged at 2000× *g* for 20 min (Combi-514R; Hanil Science Industrial, Incheon, Korea), and the supernatant was filtered using Whatman No. 1 filter paper (Whatman, Buckinghamshire, UK). The volume of the collected liquid was adjusted to 100 mL by adding distilled water.

The total soluble sugar content in the mealworms was analyzed using the phenol-sulfuric acid colorimetric method [17]. One milliliter of extract was mixed with 1 mL of 5% phenol solution and 5 mL of sulfuric acid, and it was placed at 20 °C for 30 min. The absorbance was detected at 470 nm using a spectrophotometer (Optizen 2120UV; Mecasys, Daejeon, Korea). The concentration of total soluble sugar was calculated using a standard curve constructed using D-(+)-glucose data (Sigma-Aldrich Corporation, St. Louis, MO, USA).

The content of reducing sugar in the mealworms was determined using the dinitrosalicylic acid reagent following the method described by Miller [18]. One milliliter of the extract and 1 mL of dinitrosalicylic acid agent were mixed and kept at 90 °C for 15 min. The solution was immediately cooled on ice, and its absorbance was detected at 570 nm (Optizen 2120UV; Mecasys, Korea). The concentration of reducing sugar was calculated by comparing the result with a standard curve of D-(+)-glucose.

### 2.3. Free Sugar Composition

The free sugar composition of the mealworms was determined using the modified method described by Kim et al. [19]. Mealworm powder (1 g) was mixed with 20 mL of 80% ethanol solution and extracted at 40 °C for 1 h, followed by centrifugation at 2000× *g* for 20 min (Combi-514R; Hanil Science Industrial, Korea). The supernatant was filtered with a 0.2 μm syringe filter (Advantec MRF; Advantec, Tokyo, Japan). A column (3.9 × 300 mm, 10 μm; WAT084038; Waters, Milford, MA, USA) for carbohydrate analysis was used with a PU-2089 plus high-performance liquid chromatography (HPLC) system (Jasco, Tokyo, Japan). The mobile phase constituted a mixture of acetonitrile and water (87:13, *v*/*v*) with a flow rate of 1.2 mL/min, and the isocratic system was used for mobile phase. The injection volume was 20 μL, and a refractive index (RI) detector was used for detection. The concentration of each sugar was calculated using the standard curves of common monosaccharides (glucose, fructose, and galactose; Sigma-Aldrich, St. Louis, MO, USA) and disaccharides (sucrose, maltose, and lactose; Sigma-Aldrich, St. Louis, MO, USA). The R^2^ of standard curves of mono- and di-saccharides were over 0.9999.

### 2.4. Total Glucosamine Content

The total glucosamine content of the mealworm powder was determined according to the methods described in previous studies [20,21]. Here, 30 mL of 2 M HCl was added to 5 g of mealworm powder and boiled at 95 °C in a reflux tube for 24 h. The solution was neutralized using 1 M NaOH solution, and its volume was adjusted to 100 mL by adding distilled water. One milliliter of the solution was mixed with acetylacetone solution (acetylacetone in 0.5 N sodium carbonate, 1:50, *w*/*w*) and reacted at 95 °C for 10 min. Then, 1 mL of dimethylaminobenzaldehyde solution (0.8 g of p-dimethylaminobenzaldehyde in 30 mL of ethanol and 30 mL of HCl solution) was added and the solution was maintained at 75 °C for 30 min. After cooling on ice, the volume of the solution was adjusted to 10 mL using ethanol. The absorbance of the final solution was measured at 530 nm using a spectrophotometer (Optizen 2120UV; Mecasys, Korea). The complete reaction procedure was similarly performed using a D-glucosamine standard (Sigma-Aldrich, St. Louis, MO, USA), and its standard curve was used to calculate the total glucosamine content in the mealworms.

### 2.5. Extraction of Chitin and Preparation of Chitosan from Mealworms

To remove oils from the mealworm powder, n-hexane was mixed with the powders (1:5, *w*/*v*) and the oils were extracted in a shaker at 170 rpm for 6 h (SI600R; Lab Companion, Daejeon, Korea). The liquid was separated by filtration using a filter paper (Whatman No. 1; Whatman, UK), and the same procedures were performed twice for the remaining powders. The defatted powders were dried in a hood at 20 °C for 24 h, and further dried using a speed vacuum evaporator (Maxivac alpha; Labogene ApS, Lynge, Denmark).

Chitin was extracted from defatted mealworm powders [22,23]. For protein degradation, 20 g of defatted powder was mixed with 400 mL of 1.25 M NaOH solution and maintained at 80 °C for 24 h. The solution was filtered using the ashless Whatman No. 5 filter paper (Whatman, UK) and rinsed with distilled water until neutralized, and the remaining powders were freeze-dried. The dried powders were mixed with 1.5 M HCl solution (1:10, *w*/*v*) and shaken at 20 °C in a shaker (120 rpm, 6 h) (SI600R; Lab Companion, Korea). The solution was filtered using a Whatman No. 5 filter paper (Whatman, UK) and rinsed with distilled water for neutralization. The lyophilized powder was used as the chitin sample extracted from the mealworms. The yields of mealworm chitin were determined by repeating the extraction procedure thrice.

The chitosan sample was prepared from chitin powder via deacetylation. Fifty milliliters of 50% NaOH solution and 5 g of mealworm chitin were mixed and reacted at 80 °C for 4 h. The liquids were removed by filtration using a Whatman No. 5 filter (Whatman, UK) and remained powders were rinsed with distilled water to neutralize them. The freeze-dried powder was used as the mealworm chitosan sample.

### 2.6. Moisture and Crude Protein Contents 

The moisture and crude protein contents were analyzed using methods of the Association of Official Analytical Chemists (AOAC) [24]. The moisture content was determined using the oven drying method. One gram of chitin powder was placed in a drying oven at 105 °C for 12 h, and its weight was measured. The moisture content was determined by subtracting the powder weight before and after drying. The crude protein content was determined by the Kjeldahl method using an automatic Kjeldahl analyzer (KDN; NANBEI Instrument, Zhengzhou, China).

### 2.7. Color and Whiteness Index (WI)

The color values (Hunter L*, a*, and b*) of the chitin powders were measured using a colorimeter (CM-3500d; Minolta, Tokyo, Japan). The whiteness index (WI) of mealworm chitin was calculated using an equation described in a previous study [25]: WI = 100 − [(100 − L*)^2^ + (a*)^2^ + (b*)^2^]^1/2^.

### 2.8. Field-Emission Scanning Electron Microscopy (FESEM) Imaging and Energy Dispersive X-Ray Spectroscopy (EDS) Analysis

The surface images of chitin and chitosan extracted from the mealworms were obtained using FESEM (SUPRA 55VP; Carl-Zeiss-Strasse, Oberkochen, Germany), and EDS was simultaneously performed using the same instrument. α-Chitin from shrimp (Sigma-Aldrich, USA) was also analyzed as the reference sample. The chitin and chitosan samples were fixed on a stub and coated with platinum (Sputter coater BAL-TEC/SCD 005; BAL-TEC AG, Pfäffikon, Switzerland). The images of the samples were obtained at two magnifications (×300 and ×10,000). The carbon and nitrogen contents of the samples were analyzed by EDS, and the ratio of carbon and nitrogen in the samples was used to calculate the degree of acetylation (DA) of chitin and the degree of deacetylation (DDA) of chitosan using the following equations [26,27]:

DA(%) = ((C/N) − 5.14)/1.72 × 100(1)

DDA(%) = (6.857 − (C/N))/1.7143 × 100(2)

where C and N are the carbon and nitrogen contents.

### 2.9. Nuclear Magnetic Resonance (NMR)

Proton (1H) NMR analysis was conducted using a 600 MHz AVANCE 600 NMR spectrometer (Bruker, Billerica, MA, USA). The chitin and chitosan samples were dissolved in 2% deuterated acetic acid in D2O at a concentration of 2 M, and 1H NMR spectra were obtained at 70 °C [23]. Additionally, 13C NMR spectra were obtained using a 500 MHz solid-state NMR spectrometer (AVANCE II; Bruker, Billerica, MA, USA) according to the method described by Van de Velde and Kiekens [28]. The obtained NMR peaks were used for calculating the DA of chitin and DDA of chitosan using the following formulas [29,30]:

DA(%) = (100 × I[CH_3_])/((I[C1] + I[C2] + I[C3] + I[C4] + I[C5] + I[C6])/6) × 100 (3)

where I is the intensity of each peak and C represents the carbon atoms in the chitin monomer.

DDA(%) = H1D/(H1D + HAc/3) × 100(4)

where H1D is the integral of the peak of proton H1 of the deacetylated monomer and HAc corresponds to the peak of the three protons of the acetyl group.

### 2.10. Fourier Transform Infrared Spectroscopy (FT-IR)

FT-IR spectroscopy was performed using a Nicolet 6700 spectrometer (Thermo Scientific, Waltham, MA, USA) to determine the chemical structural characteristics of chitin and chitosan obtained from the mealworms. The samples were mixed with potassium bromide and placed in a ZnSe/diamond disc. The analyzed frequency range was 4000–650 cm^−1^ and the resolution was 8 cm^−1^ [23]. The DDA of the chitosan sample was calculated using the following equation [31]: DDA (%) = 100 − (A_1655_/A_3450_) × 115.

### 2.11. X-Ray Powder Diffraction (XRD)

XRD analysis was performed using a D8 ADVANCE instrument with DAVINCI (Bruker, Madison, WI, USA) according to the method described by Yen et al. [32]. The Cu radiation (40 kV, 40 mA) was used as the X-ray source with an LYNXEYE XE detector. The collected scanned data range of 2θ was 5–50°. The crystallinity index of the chitin samples was calculated using the following formula [33]:

Crystallinity index (%) = [Ic/(Ic + Ia)] × 100(5)

where Ia is the intensity at 2θ = 16° and Ic is the intensity at 2θ = 20°.

### 2.12. Anti-Inflammatory Effect in Murine Macrophage Cell Line

The anti-inflammatory effect of mealworm chitosan was tested with the lipopolysaccharide (LPS)-induced murine macrophage cells (RAW 264.7). RAW 264.7 cells were obtained from American Type Culture Collection (ATCC, Rockville, MD, USA) and maintained in Dulbecco’s modified Eagle’s medium (DMEM) with 10% fetal bovine serum and 1% penicillin/streptomycin solution. The cells were incubated in an incubator with 5% CO_2_ at 37 °C. The cells were seeded at a concentration of 5 × 10^4^ cells per well in 96-well plates and maintained for 24 h. After removing the culture media and rinsing with phosphate buffer saline (PBS), 50 μL of mealworm chitosan-containing media was added to each well. Fifty microliters of LPS-containing media (2 μg mL^−1^) was added after an hour, and incubated for 24 h in an incubator. Then, the supernatant of each well was mixed with the same volume of Griess reagent. After reaction for 20 min, the absorbance of the solution was measured at 550 nm using a microplate reader (SpectraMax 190; Molecular Devices, Sunnyvale, CA, USA).

### 2.13. Statistical Analysis

The data are presented as mean ± standard deviation (SD) based on triplicate measurements. After normality and homogeneity tests for data, the Student’s *t*-test or one-way analysis of variance (ANOVA) was conducted using the IBM SPSS statistics v.25 software (IBM Inc., Armonk, NY, USA). Duncan’s multiple-range test was also performed for the post-hoc analysis of ANOVA (*p* < 0.05). 

## 3. Results and Discussion

### 3.1. Carbohydrate Content and Its Composition in Mealworms

While carbohydrates are the major components of most foods, animal foods have abundant proteins and lipids but low carbohydrate contents. Accordingly, the crude carbohydrate content in dried mealworms was 11.45 ± 0.38%, implying that the undried mealworms contained approximately 3.4% carbohydrate (Table 1). Among the total carbohydrates in the mealworms, the total soluble sugar content was 3.22 ± 0.10%. To further analyze the soluble sugar composition in the mealworms, the representative mono- or di-saccharide contents (glucose, fructose, galactose, maltose, sucrose, and lactose) were determined by HPLC analysis. Galactose, maltose, and lactose were not detected, and the contents of glucose, fructose, and sucrose were 31.02 ± 1.95, 77.36 ± 0.35, and 12.46 ± 0.94 mg/100 g dry basis, respectively. The glucose content of raw mealworms was approximately 12 mg/100 g, which was approximately a quarter of that determined in chicken breast [34,35] and 3–6% of that in pork [35,36]; in contrast, the glucose content of mealworms was similar to that found in fish (18–72 mg/100 g) [37]. Although free sugars are soporific chemical compounds in various ingredients and their content is associated with taste, particularly sweetness, the total free sugar content of the mealworms (120.84 ± 1.07 mg/100 g dry basis) was too low to affect the taste because the threshold of sugar is known to be approximately 0.125% [38]. Therefore, the savory taste of mealworms can be affected by the abundant free amino acids or peptides rather than the sugars [16].

Additionally, a considerable gap was observed between the contents of crude carbohydrates and total soluble sugars in the mealworms; therefore, mealworm bodies were predicted to consist of abundant insoluble carbohydrates. Chitin and its derivatives are typical insoluble carbohydrates that are found in the insect integument, and 4.84 ± 0.43% of glucosamine (the monomer of chitin) is present in dried mealworm powder. This allows the consumers to intake abundant chitins upon eating mealworms. Moreover, mealworm chitins can be applied for mass production in industries because these are easy to rear on a large scale. Therefore, chitins were extracted from the mealworms and their overall characteristics were examined to verify their availability.

### 3.2. Yield, Moisture and Crude Protein Contents, and Color Traits of Chitin Extracted from Mealworms

Common chitin products in industries constitute those originating from crustaceans such as shrimp and crab. Because of their tough textures and difficult digestion, their shells are dumped as waste; therefore, this waste is typically used for preparing chitins. Chitin and chitosan can be employed as therapeutic agents, pharmaceutical carriers, and packaging materials as natural biopolymers [39]. When chitin was extracted from mealworm powder on a laboratory scale, its yield was 4.72 ± 0.21 g/100 g dried mealworm (Table 2). The extraction may have performed properly because this amount was similar to the total glucosamine content of the mealworms (4.84 ± 0.43%); moreover, its yield was analogous to that reported by Song et al. [40] (4.91%), but considerably lower than that reported by Siregar and Suptijah [41] (10–13%). In a subsequent study, the authors reported a high yield for chitin preparation; however, we found that the color of their chitin samples was darker, and the DA (70–80%) was significantly lower than that observed in the present study. Moreover, the authors reported a high nitrogen content, which indicated an incomplete deproteinization process; therefore, it was expected that their yield could also be decreased provided sufficient purification was performed. The yield of chitin from the mealworms was significantly lower than that obtained from crustacean sources such as shrimp (approximately 20%) and crab (30–40%) [22,42], and slightly lower than that obtained from imago edible insects such as grasshoppers (4.71–11.84%) or crickets (approximately 8.7%) [43,44]. The differences between these edible insects are attributed to their growth stages because grasshoppers and crickets are adult insects that have well-developed cuticles. However, the chitin yield of mealworms was higher than that of the silkworm pupa (one of the less developed edible insects with a chitin yield of 2.5–4.2%) [45,46]. The color values of mealworm chitin were 82.21 ± 0.72, 1.81 ± 0.08, and 8.68 ± 0.37 for lightness, redness, and yellowness, respectively, whereas the color values of chitin from the crabs were 55.4–62.4, 0.3–1.1, and 14.7–16.8 for lightness, redness, and yellowness, respectively [32]. As mealworm chitin had a significantly higher lightness value but lower yellowness, it could exhibit white color compared to the chitin originating from crabs. Similarly, the WI value of mealworm chitin (80.13 ± 0.81) was significantly higher than that of the chitin from crabs (43.9–59.6) [32], and its bright color can be potentially applicable in industries.

### 3.3. Surface Microstructures of Chitin and Chitosan Derived from Mealworms

**Figure 1:**
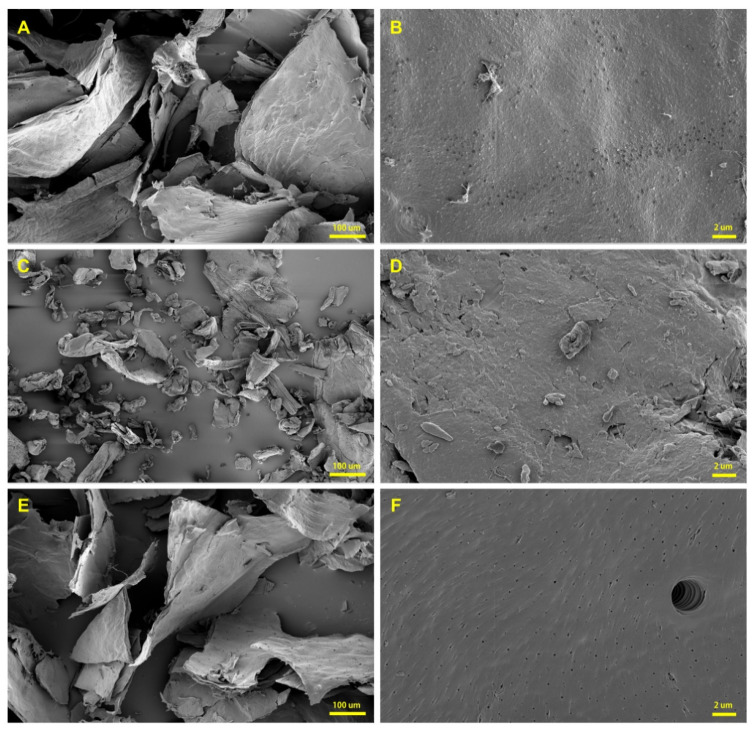
Field emission scanning electron microscopy (FESEM) data of chitin and chitosan samples. Images of mealworm chitin (**A**,**B**) (×300 and ×10,000), shrimp chitin (**C**,**D**) (×300 and ×10,000), and mealworm chitosan (**E**,**F**) (×300 and ×10,000).

The surface microstructural images of mealworm chitin and chitosan samples were obtained using FESEM (Figure 1), and commercial α-chitin from shrimp (Sigma-Aldrich, St. Louis, MO, USA) was used as the reference. One of the prominent structures of the chitin matrix is the lamellar structure that develops by the arrangement and cross-linking of chitin polymers [47], and this structure was observed in both the mealworm chitin and shrimp chitin at ×300 magnification. At a higher magnification (×10,000), the surfaces of the chitins were slightly bumpy, but chitosan showed a smooth surface with multiple pores on it. The distinct polyporous structure of chitosan was also reported in a prior study [45]. The particle size of mealworm chitin was significantly larger than that of shrimp chitin, likely due to the fine milling of shrimp chitin during commercial processing. The DA of chitin and DDA of chitosan are important indicators that represent their physical characteristics, particularly their solubility, and a simple method for measuring these involves determining the ratio of carbon and nitrogen in various substances. Thus, the carbon and nitrogen ratios of the samples were determined using the EDS system of the FESEM instrument, and the results are included in Table 3. The DA of mealworm chitin was 95.02 ± 0.22% and no significant difference was observed compared to the DA of shrimp chitin (94.81 ± 0.05%), and its value was similar to that reported in a previous study (90–98%) [23]. The DDA of the mealworm chitosan sample was 94.69 ± 0.06% as calculated using the ratio of carbon and nitrogen (5.2237 ± 0.0010).

### 3.4. Determination of Precise DA and DDA of Mealworm Chitin and Chitosan Using NMR

**Figure 2:**
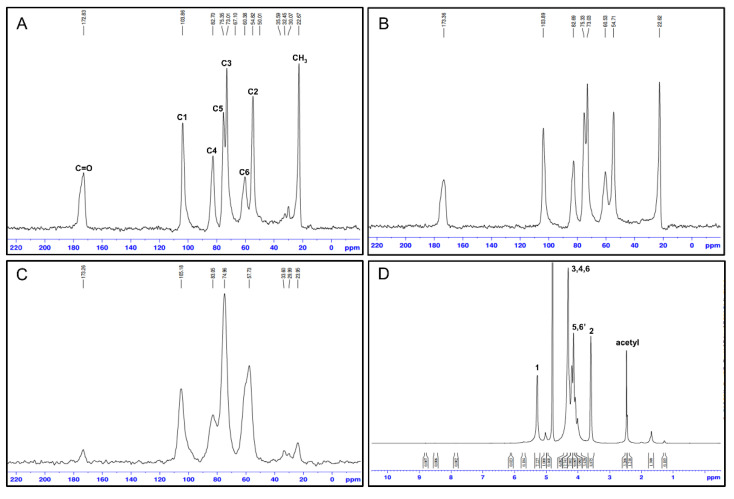
Nuclear magnetic resonance (NMR) data of chitin and chitosan. ^13^C NMR data of mealworm chitin (**A**), shrimp chitin (**B**), and mealworm chitosan (**C**). ^1^H NMR data of mealworm chitosan (**D**).

NMR is a reliable method for verifying the DA and DDA of chitin derivatives, which can assist in the determination of their structural properties [28]. Therefore, solid-state 13C NMR was used for the analyses of chitin and chitosan samples, and 1H NMR was used to analyze mealworm chitosan (Figure 2). In a study by Heux et al. [48], the chemical shifts for the commercial chitins in the 13C solid-state NMR spectra were observed at 103.5–104.7 ppm (C1), 55.2–57.6 ppm (C2), 73.3–75.0 ppm (C3), 82.4–83.0 ppm (C4), 74.7–75.7 ppm (C5), 60.1–61.8 ppm (C6), and 22.8–23.2 ppm (CH3), and these data were consistent with the results of the present study. Interestingly, although the major chemical shift patterns were analogous for mealworm chitin and shrimp chitin, a distinctive chemical shift was observed at 30 ppm only in the data for the mealworm chitin. Zhang et al. [49] reported that this chemical shift was attributed to the catechol moiety, which is a hydroxyl-containing aromatic compound [50]. In the cuticle layer of the insects, catechol is a vital constituent that forms cross-linking networks with other structural polymers [51]. Thus, the presence of a peak at 30 ppm indicated that mealworms contained catechol molecules in their body, but this was not observed in the shrimp chitin. Meanwhile, the DA values calculated using NMR were 98.3% for mealworm chitin and 97.8% for shrimp chitin, and their values were slightly higher than the values calculated using the carbon and nitrogen ratio. This difference could be caused by the adulteration of proteins or other matter, but the prepared mealworm chitin showed a DA value similar to that of the commercially obtained shrimp chitin. Thus, it was expected that mealworm chitin could show physical characteristics similar to those of the shrimp chitin. The DDA of mealworm chitosan was calculated based on the 1H NMR data (89.4%), and the range for this value was reported in a previous study (83–93%) in which mealworm chitosan was prepared [32]. The DDA of mealworm chitosan was higher than that of the commercial chitosans derived from crustaceans (70–85%) [52].

### 3.5. FT-IR Spectral Analyses of Mealworm Chitin and Chitosan

**Figure 3:**
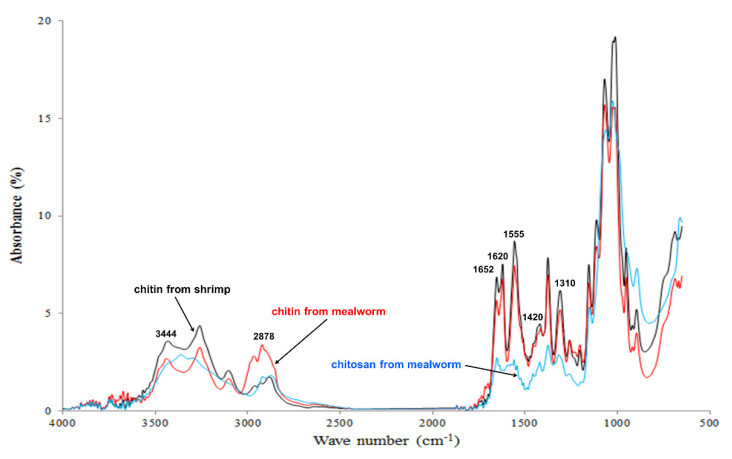
Fourier transform infrared spectroscopy (FT-IR) data of chitin and chitosan.

The FT-IR spectra of the samples are shown in Figure 3, and the relevant absorbance peaks have been reported in a prior study [28], including the signals for hydroxyl stretching (3450 cm^−1^), C–H stretching (2878 cm^−1^), C–H deformations (1420 cm^−1^), C=O stretching in secondary amide (1550, 1625, 1655, and 1661 cm^−1^), and C–N stretching in secondary amide (1320 cm^−1^). Mealworm chitin shows a spectral pattern similar to that of shrimp α-chitin. The chitin fibers are commonly classified into three types (α-, β-, and γ-chitin) based on their crystal structures, and each type shows different physical properties. The chitin polymer is generally arranged in parallel, and α-chitin has an inverse configuration between the adjoined chitin chains, but β-chitin consists of equally aligned chitin fibers [53]. There are several distinguishing signals of β-chitin in comparison to those of α-chitin. First, separate peaks for the bands at 1660 cm^−1^ (corresponding to the hydrogen bond between C=O and NH groups) and 1625 cm^−1^ (corresponding to the hydrogen bond between C=O and HOCH2 groups) are not observed, and β-chitin shows a peak at 1430 cm^−1^ instead of 1416 cm^−1^ [54,55,56]. As mealworm chitin does not show spectral features of β-chitin but instead the standard FT-IR spectral patterns of α-chitin, the main chitin type in mealworms is identified as α-chitin. In contrast, mealworm chitin shows a relatively high absorbance at 2878 cm^−1^ (C–H stretching) and low absorbance at 3444 cm^−1^ (hydroxyl stretching) compared to the shrimp α-chitin. These distinct features are attributed to dibutyrylchitin (DBC), where chitin contains bonds of butyryl groups at the C3 and C6 positions [28,57]. DBC is typically obtained by the artificial conversion of chitins, and it has high solubility, suitability for fabricating films, and superior biodegradability [58]. While DBC is rarely found in natural sources, its distinct FT-IR spectral peaks (2878 and 3444 cm^−1^) have been detected in the data for the chitin extracted from silkworm pupa, but it shows a significantly smaller peak than that observed in the present study [45]. Hence, DBC may be naturally contained in the chitin obtained from insects, and the mealworms show a notable signal; however, further confirmation is still required.

### 3.6. Determination of Crystalline Structures of Mealworm Chitin and Chitosan by XRD

**Figure 4:**
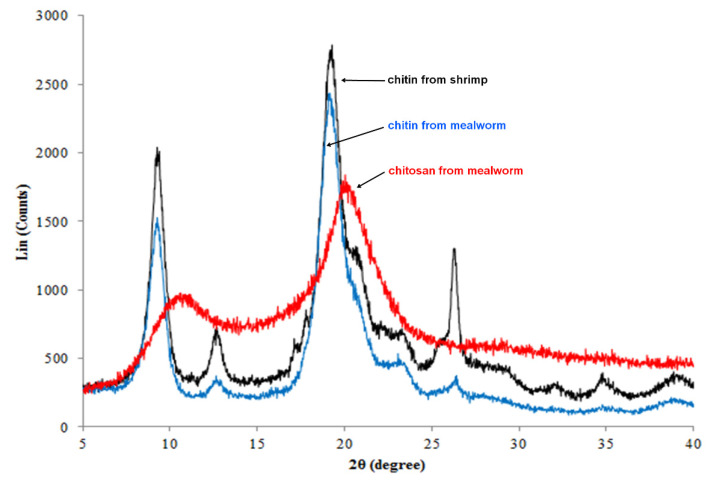
X-ray diffraction (XRD) data of chitin and chitosan.

To verify the crystalline structural characteristics of the chitin samples, XRD analysis was conducted (Figure 4). The α-chitin peaks were expected to be observed at 9.6°, 19.6°, 21.1°, 23.7°, and 36°, which were observed for chitins obtained from mealworm and shrimp [23]. In addition, other insect species (e.g., grasshopper, wasp, cockroach, and cicada) showed similar XRD patterns in the reported studies; therefore, α-chitin constitutes the prime structure of insect chitin [59,60]. It is well-known that the antiparallel arrangement of the chitin fibers of α-chitin forms a lamellar structure, which is observed in the SEM data obtained in this study. The intensities of the peaks at 26.3° and 12.7° in the data for mealworm chitin were lower than those of shrimp chitin, and this trend was also observed for the chitin obtained from silkworm pupa [49]. The XRD pattern of DBC was observed, as DBC showed clear peaks at approximately 9.6° and 21.1° but the peaks at 12.7° and 26.3° were not present [61]; therefore, the presence of DBC could decrease the corresponding peaks (12.7° and 26.3°). The crystallinity indices of chitin samples were 57.85 ± 0.17% and 62.04 ± 0.10% for mealworm and shrimp, respectively (Table 4). The crystallinities of silkworm pupa and beetle larvae were 54% and 58%, respectively [49], and their values were similar to that of mealworms (57.85%) observed in the present study. In contrast, the crystallinity indices of crustacean chitins had higher values (60–70%) than those of the mealworm chitin [62] as mealworms have a less firm shell. The tender structure is advantageous during the preparation of soft chitin materials because the crystallinity of chitin is associated with hardness [63]. Therefore, the use of mealworm chitin can have benefits in such specific applications.

### 3.7. NO Reduction Activity of Mealworm Chitosan

**Figure 5:**
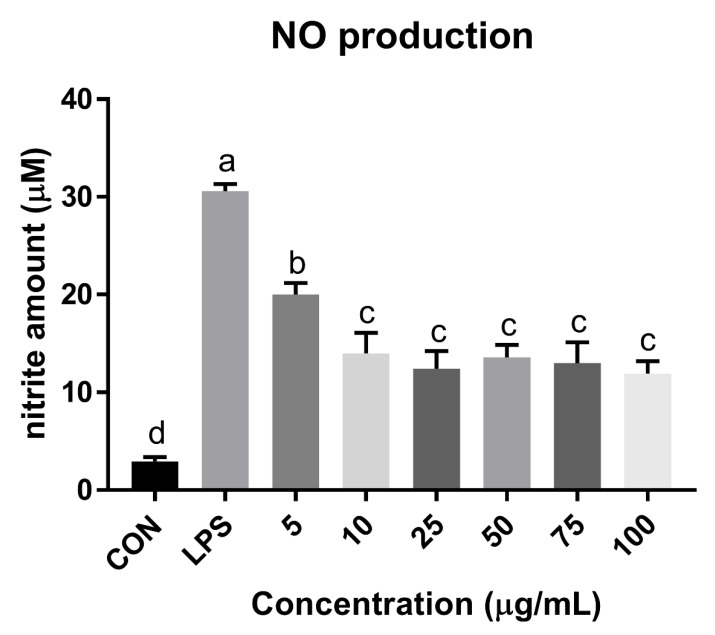
Nitric oxide reduction effects of mealworm chitosan on lipopolysaccharide (LPS)-stimulated murine macrophage cell line (RAW 264.7). Different superscripts (a–d) indicate significant differences at *p* < 0.05.

The anti-inflammatory and anti-degenerative arthritis activities of glucosamine and chitosan are well known, but the efficacy of mealworm chitosan has not been sufficiently proven [64,65]. To verify this, mealworm chitosan was treated at various concentrations in the LPS-induced RAW 264.7 cell line assay (Figure 5). Mealworm chitosan showed notable NO reduction effect on the macrophage cells, and its efficacy was relatively higher than that reported in other studies that used chitosan obtained from other animal sources [66,67]. In addition, the anti-inflammatory effect was attributed to not only chitosan, but also peptides, proteins, and unsaponifiable matter of the oils from mealworms in prior studies [16,66,67]. Hence, mealworm chitosan and mealworm-derived products can potentially be used for therapeutic applications in inflammatory disorders.

## 4. Conclusions

The aim of this study was to determine the carbohydrate content and composition of mealworms as well as examine the characteristics of mealworm chitin and chitosan because chitin derivatives are the major carbohydrate constituents in mealworms. The mealworms had a low carbohydrate content (11.5%), while the soluble sugar content was only 3.2%. Instead, chitins constituted almost half of the total carbohydrate content in the mealworms. The prepared mealworm chitin exhibited a white flake form, and its yield was 4.7 g per gram of dried mealworm. The prominent structural type of mealworm chitin was α-chitin, similar to that observed for other insects, and it developed lamellar structures in the matrices. The calculated DA of chitin and DDA of chitosan were 98.3% and 89.4%, respectively, based on NMR analysis, which were analogous to those of the commercial products. As the DA and DDA values are closely related to the physical properties of the chitin derivatives and those of mealworms showed similar values, mealworm chitin derivatives could replace the commercial chitins without considerable changes in the product quality. The crystallinity index of mealworm chitin was relatively lower than that of the crustacean-derived chitins, indicating that mealworm chitin could be applied to the products with soft textures. Considering the FT-IR and XRD patterns, it was assumed that DBC was compounded with α-chitin in the mealworms, and this could be highly beneficial because DBC showed superior properties such as high solubility, suitability for fabricating films, and superior biodegradability; however, it has been rarely found in natural sources. In addition, mealworm chitosan showed excellent anti-inflammatory effects in the LPS-induced murine macrophage cell line. Therefore, the physical characteristics of mealworm chitin and chitosan are expected to be suitable for use in industries, and afford additional advantages such as tender texture and potent anti-inflammatory effects.

## Figures and Tables

**Table 1:** Carbohydrate content and its composition in dried mealworm.

	Dried Mealworm
Crude carbohydrate (g/100 g)	11.45 ± 0.38
Total soluble sugar (g/100 g)	3.22 ± 0.10
Reducing sugar (g/100 g)	0.19 ± 0.01
Total glucosamine (g/100 g)	4.84 ± 0.43
Free sugar (mg/100 g)	
Glucose	31.02 ± 1.95
Fructose	77.36 ± 0.35
Sucrose	12.46 ± 0.94
Total	120.84 ± 1.07

Data are expressed as mean ± SD.

**Table 2:** Yield, moisture and protein contents and color characteristics of the extracted mealworm chitin.

	Chitin from Mealworm
Yield (g chitin/100 g dried mealworm powder)	4.72 ± 0.21
Moisture (g/100 g chitin)	2.38 ± 0.05
Crude protein (g/100 g chitin)	9.96 ± 0.36
Color	
L* (lightness)	82.21 ± 0.72
a* (redness)	1.81 ± 0.08
b* (yellowness)	8.68 ± 0.37
Whiteness index (WI)	80.13 ± 0.81

Data are expressed as mean ± SD.

**Table 3:** C/N ratio determined based on energy dispersive X-ray spectroscopy (EDS) data and the predicted degree of acetylation for chitin or degree of deacetylation for chitosan.

	Chitin from Mealworm	Chitin from Shrimp	*t*-Value
C/N ^†^	6.7743 ± 0.0038	6.7707 ± 0.0010	−1.310 ^NS^^§^
DA ^‡^ (%)	95.02 ± 0.22	94.81 ± 0.05	

Data are expressed as mean ± SD. ^†^ C/N: ratio of carbon and nitrogen elements. ^‡^ DA: degree of acetylation. ^§NS^: Not significant.

**Table 4:** Degree of crystallinity of extracted chitin from mealworm or shrimp.

	Mealworm Chitin	Shrimp Chitin	*t*-Value
Degree of crystallinity (%)	57.85 ± 0.17	62.04 ± 0.10	−37.053 ***

Data are expressed as mean ± SD. *** Significant difference at *p* < 0.001.

## Data Availability

Not applicable.
